# Biomonitoring of Exposure to Urban Pollutants and Oxidative Stress during the COVID-19 Lockdown in Rome Residents

**DOI:** 10.3390/toxics10050267

**Published:** 2022-05-21

**Authors:** Flavia Buonaurio, Francesca Borra, Daniela Pigini, Enrico Paci, Mariangela Spagnoli, Maria Luisa Astolfi, Ottavia Giampaoli, Fabio Sciubba, Alfredo Miccheli, Silvia Canepari, Carla Ancona, Giovanna Tranfo

**Affiliations:** 1 Department of Chemistry, Sapienza University of Rome, 00185 Rome, Italy; flavia.buonaurio@uniroma1.it (F.B.); francesca.borra@libero.it (F.B.); marialuisa.astolfi@uniroma1.it (M.L.A.); 2 Department of Occupational and Environmental Medicine, Epidemiology and Hygiene, INAIL, Monte Porzio Catone, 00144 Rome, Italy; d.pigini@inail.it (D.P.); e.paci@inail.it (E.P.); m.spagnoli@inail.it (M.S.); 3 Department of Environmental Biology, Sapienza University of Rome, 00185 Rome, Italy; ottavia.giampaoli@uniroma1.it (O.G.); fabio.sciubba@uniroma1.it (F.S.); alfredo.miccheli@uniroma1.it (A.M.); silvia.canepari@uniroma1.it (S.C.); 4 NMR-Based Metabolomics Laboratory (NMLab), Sapienza University of Rome, 00185 Rome, Italy; 5 Department of Epidemiology, Lazio Regional Health Service, 00154 Rome, Italy; c.ancona@deplazio.it

**Keywords:** benzene, COVID-19 pandemic, lockdown, Rome, elements, urban traffic, oxidative stress, metabolomics

## Abstract

Background: The objective of this study is to evaluate the effects of traffic on human health comparing biomonitoring data measured during the COVID-19 lockdown, when restrictions led to a 40% reduction in airborne benzene in Rome and a 36% reduction in road traffic, to the same parameters measured in 2021. Methods: Biomonitoring was performed on 49 volunteers, determining the urinary metabolites of the most abundant traffic pollutants, such as benzene and PAHs, and oxidative stress biomarkers by HPLC/MS-MS, 28 elements by ICP/MS and metabolic phenotypes by NMR. Results: Means of s-phenylmercaputric acid (SPMA), metabolites of naphthalene and nitropyrene in 2020 are 20% lower than in 2021, while 1-OH-pyrene was 30% lower. A reduction of 40% for 8-oxo-7,8-dihydroguanosine (8-oxoGuo) and 8-oxo-7,8-dihydro-2-deoxyguanosine (8-oxodGuo) and 60% for 8-oxo-7,8-dihydroguanine (8-oxoGua) were found in 2020 compared to 2021. The concentrations of B, Co, Cu and Sb in 2021 are significantly higher than in the 2020. NMR untargeted metabolomic analysis identified 35 urinary metabolites. Results show in 2021 a decrease in succinic acid, a product of the Krebs cycle promoting inflammation. Conclusions: Urban pollution due to traffic is partly responsible for oxidative stress of nucleic acids, but other factors also have a role, enhancing the importance of communication about a healthy lifestyle in the prevention of cancer diseases.

## 1. Introduction

In the year 2020, there was a viral pandemic known as COVID-19 (Coronavirus Disease-2019), generated by the SARS-CoV-2 virus (Severe Acute Respiratory Syndrome- Coronavirus 2) still ongoing, to varying degrees in almost all countries [1].

According to WHO, there were 4,251,460 confirmed cases and 84,997 deaths worldwide on December 28th 2020, of which 102,418 cases and 3358 deaths were registered in Italy on the same date [2].

The first two Italian cases were confirmed on 30 January 2020 [3]; the subsequent outbreaks detected in northern and central Italy led to a series of restrictions, such as local quarantines, but with the increase in infections, new stricter restriction measures were gradually introduced and extended to the entire national territory [4].

On 9 March 2020, a national lockdown started, during which face-to-face teaching in schools and universities was suspended and working from home was promoted wherever possible; travel for unnecessary reasons, sports activities, demonstrations and events were prohibited, and museums and theatres were closed; non-essential retail commercial activities, catering services, religious celebrations, and gatherings of people in public places were suspended by law [4].

The restrictive measures were extended several times until 3 May 2020, until they were almost completely removed, starting from 15 June 2020 [5].

In addition to the various social, economic and political consequences [6,7], the effects of the lockdown were also evident at the environmental level, especially in terms of a drastic decrease in vehicular traffic, whose effects were visible above all in large urban centers, such as Rome [8] and Milan [9]. A reduction in emissions was also measured in China [10].

The lockdown scenario was a unique condition, which rendered possible the direct measurement of the effects of reduced exposure to traffic pollution on the same group of subjects for a long period [11]. Therefore, this study is the first one that aims to investigate the reduction in exposure biomarkers related to traffic pollution in Rome, due to the restrictions imposed by the Government to contain the COVID-19 pandemic.

Traffic-related air pollution is a global concern due to the adverse health effects on humans [12]. Both short- and long-term exposure to air pollutants increase the risk of respiratory and cardiovascular diseases in the population [13]. Outdoor air pollution is an established risk factor for lung cancer incidence and mortality [14]. Among traffic-related air pollutants, there are benzene, polycyclic aromatic hydrocarbons (PAHs) and toxic metals [15,16,17]. Benzene and PAHs can penetrate biological membranes and generate reactive oxygen species (ROS), which can affect inflammation associated with immune responses [18,19]. Benzene is a known carcinogen that causes hematotoxicity even at exposure levels below 1 ppm [20,21] and has been associated with nucleic acid oxidation biomarkers [22]. It has an influence on the central nervous system and it is involved in metabolic function, such as insulin resistance [23]. Furthermore, benzene is reported to be the cause of myeloid leukemia, myelodysplastic syndrome and a probable cause of non-Hodgkin lymphoma [24]. For this reason, the Directive 2008/50/EC on ambient air quality and cleaner air for Europe has set a limit value at 5 µg/m^3^ of airborne benzene as the annual average since January 2010 [25]. Polycyclic aromatic hydrocarbons (PAHs) are also a class of carcinogenic agents [26]. Their origin is the incomplete combustion and pyrolysis of organic materials, especially wood, coal, oil and waste. PAHs containing four rings or less typically remain in a gaseous form when released into the atmosphere, particularly benzo[a]pyrene, which is classified by IARC (International Agency for Research on Cancer, Lyon, France) as a class 1 carcinogen. The PAH biomarkers 1-hydroxypyrene (1-OHPy), 6-hydroxynitropyrene (6-OHNPy) and 3-hydroxybenzo[a]pyrene (3-OHBaPy) have been demonstrated to correlate well with oxidative stress biomarkers [27]. Short-term exposure to PAHs has been reported to cause impaired lung function with asthmatics and thrombotic effects in people with coronary heart disease. Furthermore, exposure to high levels of PAHs can generate acute health effects, such as eye irritation, nausea, vomiting, diarrhea and confusion. In addition, skin, lung, bladder and gastrointestinal cancers have been reported as major long-term effects of PAH exposure [28].

Both essential and non-essential elements, including heavy metals, have also been detected in urban air particulate matter (PM) [16]. The composition and content of heavy metals vary greatly among atmospheric particles of different sizes and origins [16]. Vehicular traffic is one of the major sources of heavy metals in urban areas [29]. In road dust, contributions are due to worn out tires, friction and brake discs and to the wear of the road surface [30]. The most widespread and documented elements include cadmium (Cd), cobalt (Co), chromium (Cr), copper (Cu), mercury (Hg), nickel (Ni), lead (Pb), antimony (Sb) and zinc (Zn) [17,31,32,33,34]. Toxic elements in road dust enter the human body mainly via three routes: direct inhalation, ingestion and dermal contact [35,36,37,38]. In addition, road dust can be resuspended by people walking or cycling and by the vehicles themselves, resulting in greater exposure levels around the road [32]. Toxic elements can affect human health, causing DNA damage, with induces mutagenic, teratogenic and carcinogenic effects [39,40,41]. Therefore, human exposure to road dust may increase health risks, including carcinogenic contamination [42,43]. Urinary metals were positively associated with oxidative stress biomarkers at exposure levels relevant to the general population [44].

The objective of this study is to evaluate the effects of traffic on human health comparing biomonitoring data measured during the COVID-19 lockdown, when restrictions led to a drastic reduction in road traffic in Rome, with the same parameters measured in 2021. Therefore, the exposure to benzene, PAHs and 28 elements was assessed by the biological monitoring of urine samples collected during the lockdown months (March–April 2020) and in the same period of the following year, by a group of 47 Rome resident volunteers, who worked from home during 2020. The exposure biomarkers were correlated to effect biomarkers, namely the urinary oxidative stress biomarkers produced by the oxidation of guanine residues in DNA and RNA [45].

1-hydroxynaphtalene (1-OHNAP) and 2-hydroxynaphtalene (2-OHNAP) are the metabolites of naphthalene, the most abundant PAHs in human urine. Urinary 1-OHPy is the biomarker of occupational exposure to polycyclic aromatic hydrocarbons mixtures. The major urinary metabolite of benzo[a]pyrene, 3-OHBaPy, has been considered closely related to genotoxicity [46]. 1-nitropyrene has been used as a molecular marker for diesel exhaust gases, which significantly contributes to particulate-associated toxicity [47]. Urinary metabolites of nitropyrene have been evaluated for their usefulness as markers of short-term exposure to diesel exhaust [48].

Cotinine is the main metabolite of nicotine and a biomarker for smoking [49]. Cigarette smoking is a major source of benzene exposure in active smokers and can affect the levels of biological markers of benzene exposure in non-smokers exposed to second-hand smoke [41,50].

Oxidatively generated damage to DNA and RNA plays an important role in cancer development, cardiovascular and neurodegenerative diseases, diabetes, pulmonary fibrosis, and much more [51,52]. Guanine is the DNA base most susceptible to oxidation because of its low redox potential, leading to the formation of 8-oxo-7,8-dihydroguanine (8-oxoGua), the most common lesion, and of 8-Oxo-7,8-dihydro-20-deoxyguanosine (8-oxodGuo). 8-oxoGua and 8-oxodGuo found in human urine originate from DNA repair mechanisms and possibly also from the turnover of oxidatively damaged DNA. The oxidative modifications of RNA guanine can lead to the formation of both 8-oxo-7,8-dihydroguanine (8-oxoGua) and 8-oxo-7,8-dihydroguanosine (8-oxoGuo) [53,54]. Attacks of ROS on DNA and RNA, therefore, lead to the urinary elimination of 8-oxoGua, 8-oxodGuo and 8-oxoGuo, which are considered biomarkers of oxidatively generated damage on DNA and RNA: they are always detectable in the general population as the result of the exposure to oxidative stress agents, which can have different origins [45].

The same urine samples were also subjected to NMR untargeted metabolomic analysis: air pollutant exposures have been associated with metabolic pathways primarily related to oxidative stress and inflammation, as assessed through untargeted metabolomics in 23 studies [55].

## 2. Materials and Methods

### 2.1. Enrollment and Sampling Campaigns

The studied group consisted of 47 volunteers, 20 males and 27 females, aged 47.51 ± 17.33 years, mainly researchers and their families, working from home during the lockdown; only 2 were smokers.

Participants received and signed an informed consent and a brief questionnaire about physical characteristics (sex, age and work); a numeric code was assigned to each subject to protect their privacy. Urine samples were collected by the volunteers during the lockdown period (March–April 2020) and in the same period of the following year, and immediately frozen until being transferred to the laboratories for the analyses. All the urine samples were analyzed by liquid chromatography/tandem mass spectrometry (HPLC/MS-MS), inductively coupled plasma mass spectrometer (ICP-MS), cold vapor generation atomic fluorescence spectrometry (CV-AFS) and by proton nuclear magnetic resonance (^1^H-NMR). The urinary metabolites of benzene S-phenylmercapturic acid (SPMA), five PAHs, three biomarkers of oxidative stress to nucleic acids and 28 elements were determined, following the same pattern of previous studies on groups of general population volunteers [27,56].

The study was a non-interventional/observational study based on the definitions of the European Clinical Trials Directive 2001/20/EC, for which the approval of an ethics committee was not requested [57]; it was conducted according to the Declaration of Helsinki and followed the International Code of Ethics for Occupational Health Professionals of the International Committee of Occupational Health [58].

### 2.2. Targeted Monitoring

#### 2.2.1. Instrumentation and Chemical Supplies

The HPLC/MS-MS is an API 4000 triple-quadrupole mass spectrometry detector equipped with a Turbo Ion Spray (TIS) probe (AB Sciex, Framingham, MA, USA) coupled to a Series 200 LC quaternary pump (PerkinElmer, Norwalk, CT, USA). Detection was carried out in the multiple reaction monitoring mode (MRM) and parameters were optimized for the analytes by the automated infusion quantitative optimization procedure and then refined by flow injection analysis (FIA) using pure standards. Urine samples were stored at −25 °C and analyzed immediately after thawing. The 1.5 version of Analyst^®^ software (AB Sciex, Framingham, MA, USA) was used for instrument control. The analytical results are expressed in µg/g of creatinine. Urinary creatinine was determined by the Jaffè method using the alkaline picrate test with UV/Vis detection at 490 nm [59].

The analytical reference standards of 1- and 2-OHNaP, 1-OHPy, 6-OHNPy 3-OHBaPy, SPMA, Cotinine, 8-oxoGua, 8-oxodGuo and 8-oxoGuo were purchased by Spectra 2000 s.r.l (Rome, Italy). The isotope labelled internal standards Cotinine d_3_, 2-OHNAP d_7_, 1-OHPy d_9_, 3-OHBaPy d_11_, DL-SPMA-3,3 d_2_, (^13^C^15^N_2_) 8-oxodGuo and (^13^C^15^N_2_) 8-oxoGuo were obtained from CDN Isotopes Inc. (Pointe-Claire, QC, Canada). (^13^C^15^N_2_) 8-oxoGua (98%) was obtained from Cambridge Isotope Laboratories Inc. (Tewksbury, MA. USA); 3-NO_2_Tyr was purchased by Cayman Chemical Company (USA) and 3NO_2_Tyr d3 from TRC (Toronto, Canada). Multi-elemental standard solutions for ICP-MS analysis and Hg standard solution for CV-AFS determination were obtained from VWR International (Milan, Italy) and SCP Science (Baie D’Urfé, Quebec, Canada), respectively. For the preparation of ICP-MS internal standards, single standard solutions of In, Rh, Sc and Th (Merck KGaA, Darmstadt, Germany) and Y (Panreac Química, Barcelona, Spain) were used. 5-methylCytidine (5-MeCyt), glacial acetic acid 30%, NH_3_, dimethyl sulfoxide, sodium hydroxide solution (50–52% in water), sodium borohydride and CHROMASOLV^®^ gradient grade 99.9% methanol and acetonitrile for HPLC/MS 99.9%, carbon disulfide low benzene content, and n-exane 99.9% were obtained from Sigma Aldrich (Milan, Italy). Sodium hydroxide (98%, anhydrous pellets, RPE for analysis, ACS-ISO), hydrochloric acid (30% suprapure) and nitric acid (67% suprapure) were purchased by Carlo Erba Reagents (Milan, Italy). Purified water was obtained from a Milli-Q Plus system (Millipore Milford, MA, USA). The SPE cartridges, Sep-Pak Plus C18 (10 mL, 500 mg), were supplied by Waters (Waters S.p.A. Milan, Italy). Anotop 10LC syringe filter device (0.2 m pore size, 10 mm diameter) and syringe filter with cellulose nitrate membranes (0.45 mm pore size) were purchased from Whatman Inc. (Maidstone, UK) and GVS Filter Technology (Indianapolis, IN, USA), respectively. A Sinergi LUNA C8 column (250 × 4.6 mm, 4 μm), a Kinetex^®^ 2.6 µm Polar C18 100 Å (150 × 4.6 mm) (Torrance, CA, USA) and Discovery C18 (150 × 4.6 mm, 5 µm) provided by Merck KGaA, (Darmstadt, Germany) were used for the study.

#### 2.2.2. SPMA and Cotinine Analysis

DL S-phenylmercapturic acid (DL-SPMA), cotinine and the deuterium-labeled internal standards DL-SPMA-3,3-d_2_ and cotinine-d_3_ were determined with the following method [21]. Briefly: A total of 25 µL of internal standard (deuterated SPMA solution in methanol 1 mg/L) and 50 µL of 6N HCl were added to 5 mL of urine and, subsequently, subjected to solid-phase extraction (SPE) on Sep-pak Plus C18, 500 mg, 6 mL; 20 µL of the eluate was then analyzed using a Discovery C18, 150 × 4.6 mm reverse phase column, with a gradient of acetonitrile/methanol 90/10 *v/v* and acetic acid 0.5% *v/v* in water. The precursor→product ionic transitions monitored were in the negative ion mode. 238.1→109.1 for SPMA, and 240.1→109.1 for SPMAd_2_ and in the positive ion mode 177.3→80.10 for cotinine and 180.3→80.10 for cotinine-d_3_.

#### 2.2.3. PAH Analysis

PAH metabolites were determined following a modified version of a published method [60]: A total of 25 µL of internal standard mixture (deuterated PAH standard solution in methanol 1 mg/L), 50 µL of β-glucuronidase-arilsulfatase enzyme from Elix Pomatia and 0.5 mL of acetate buffer 0.1 M at pH 5 were added to 5 mL of urine and, subsequently, incubated in a thermostatic bath at 38 °C for 16 h to hydrolyze the PAH metabolite conjugates. After enzymatic hydrolysis, the samples were then extracted with n-exane and 20 µL injected in the HPLC/MS/MS for the analysis; a reverse phase Luna C8 250 × 4.6 (Phenomenex, Torrance, CA, USA) column was used with a gradient of methanol and water. In the negative ion mode, the precursor→product ionic transitions monitored were 217.1→189.1 for 1-OHPy and 226.0→198.1 for 1-OHPy-d_9_, 262.0→231.9 for 6-OHNPy, 267.1→239 for 3-OHBaPy and 278.0→250.0 for 3-OHBaPy-d_11_, 142.9→115.2 for 1 and 2-OHNAP, and 149.8→122 for 2-OHNAP-d7.

#### 2.2.4. Determination of Oxidative Stress Biomarkers

The urinary concentrations of 8-oxoGua, 8-oxoGuo, 8-oxodGuo and 3-NO_2_Tyr were determined according to a previously described method [61] with modifications in the sample thawing, dilution solvents, chromatographic column and mobile phases. The samples were thawed in lukewarm water at around 37 °C, vortexed and centrifuged at 10,000× *g* for 5 min; the urine supernatant was added with a mixture of mixture internal standard, ((13C15N2) 8-oxoGua, (13C15N2) 8-oxoGuo, (13C15N2) 8-oxodGuo and 3-NO_2_Tyr d3) and injected into the HPLC-MS/MS system. The reference standards of the analytes were firstly dissolved in DMSO, then in methanol and finally diluted with water. The chromatographic column was a Kinetex^®^ 2.6 µm Polar C18 100 Å (150 × 4.6 mm) and the mobile phase consisted of a gradient of a mixture of acetonitrile/methanol 90/10 *v/v* and 0.5% acetic acid in water. The same method was also used for 5-methylCytidine (5-MeCyt) and cotinine, but after diluting the sample 1:100, and after adding the internal standard (Cotinine d3), using a different chromatographic column, Discovery C18 (150 × 4.6 mm, 5 µm). The precursor/product ionic transitions monitored (positive ion mode) were 168.0→140.0 and 171.0→143.0 for 8-oxoGua and its internal standard, 284.3→168.0 and 287.13→171.1 for 8-oxodGuo and its internal standard, 300.24→168.2 and 303.24→171.0 for 8-oxoGuo and its internal standard, 226.99→181.0 and 229.99→184.0 for 3-NO_2_Tyr and its internal standard; 257.95→126.100; 180.3→80.10 was the transition monitored for 5-MeCyt, 177.3→80.10 for cotinine and 180.3→80.10 for cotinine-d3 were used as internal standard for both 5-MeCyt and cotinine.

#### 2.2.5. Determination of Urinary Elements

All elements except Hg were analyzed with a quadruple ICP-MS (820-MS; Bruker, Bremen, Germany) equipped with a concentric glass nebulizer (0.4 mL min^−1^; MicroMistTM; Analytik Jena AG, Jena, Germany) and a cyclonic spray chamber. Analyses were performed in standard mode and collision–reaction interface (CRI) mode. For As, Ca, Cr, Fe, Mn, Se and V, the assays were run in the CRI mode with He and H_2_ (99.9995% purity; SOL Spa, Monza, Italy) as cell gases. For B, Ba, Be, Bi, Cd, Co, Cs, Cu, K, Li, Mg, Mo, Na, Ni, Pb, Rb, Sb, Sn, Sr, Te, Tl and Zn, the assays were run in standard mode. A CV-AFS (AFS 8220 Titan, FullTech Instruments, Rome, Italy) was used for Hg determination. The preparations of the calibration standards and the detailed ICP-MS and CV-AFS operating conditions were described in previous papers [62,63,64]. The limits of determination (LoDs) and quantification (LoQs) were calculated, respectively, as three and ten times σ/b, where σ is the standard deviation of blank determination (ten replicates) and b is the slope of the calibration curve. Results for LoDs and LoQs were shown in Supplementary Table S1. Urine samples (1 mL) were diluted 1:5 with 3% HCl or 1:10 with 2% HNO_3_ and filtered using a syringe filter with cellulose nitrate membranes prior to CV-AFS or ICP-MS analysis, respectively.

### 2.3. Analytical Determination of Urinary Metabolic Profiles

#### 2.3.1. Sample Preparation for NMR Analysis

Untargeted biomonitoring was performed on 34 pairs of matched samples. An amount of 1200 μL of urine was centrifuged at 11,000× *g* for 15 min at 4 °C to remove the cellular debris. A total of 100 μL of a trimethylsilylpropionic-2,2,3,3-d4 acid (TSP) in D_2_O solution (2 mM final concentration) as internal standard were added to 1 mL of centrifuged samples and the pH was measured and adjusted at pH = 7 by adding small amounts of NaOH or HCl. Finally, 700 μL of sample were transferred in precision tubes for NMR experiments.

#### 2.3.2. ^1^H-NMR Spectroscopy

^1^H-NMR spectra were acquired at 298 K using a JEOL JNM-ECZR spectrometer (JEOL Ltd., Tokyo, Japan) equipped with a magnet operating at 14.09 Tesla and 600.17 MHz for 1H frequency. All the spectra were recorded with 64k points and 64 scans, setting spectral width to 9.03 KHz (15 ppm), with a pre-saturation pulse length of 2.00 s and a relaxation delay of 5.72 s, for an acquisition time of 5.81 s. The identification step was achieved by two-dimensional experiments (^1^H-^1^H Homonuclear Total Correlation Spectroscopy (TOCSY), ^1^H-^13^C Heteronuclear Single Quantum Correlation (HSQC) and Heteronuclear Multiple Bond Correlation (HMBC)) on selected samples and confirmed by literature comparison. TOCSY experiments were recorded at 298 K with a spectral width of 15 ppm in both dimensions, using 8 k × 256 data points matrix, repetition time of 3.00 s and 80 scans, with a mixing time of 80.00 ms. HSQC experiments were acquired with a spectral width of 9.03 KHz (15 ppm) in proton dimension and 30 KHz (200 ppm) in the carbon dimension, using 8 k × 256 data point matrix for the proton and the carbon dimensions, respectively, with a repetition delay of 2 s and 96 scans. One-dimensional NMR spectra were processed and quantified by using the ACD Lab 1D-NMR Manager ver. 12.0 software (Advanced Chemistry Development, Inc., Toronto, ON, Canada); 2D-NMR spectra were processed using JEOL Delta v5.3.1 software (JEOL Ltd., Tokyo, Japan). All the NMR spectra were manually phased, baseline corrected and referenced to the chemical shift of the TSP methyl resonance at δ = 0.00. The quantification of metabolites was obtained by comparing the integrals of their diagnostic resonances with the internal standard TSP integral and normalized for their number of protons. Metabolite levels are expressed as μmol mmol^−1^ of creatinine, referred at its δ = 4.05 ppm resonance.

### 2.4. Data Analysis and Statistics

The descriptive statistics of the results are presented in tables (mean and standard deviation, median and 5/95th percentiles, and minimum and maximum values). Data below LOD were substituted with LOD/2. Data were analyzed with the *t*-test on log-transformed data to determine differences between the time series. Statistical analysis was performed using IBM SPSS Statistics 25 (IBM, Armonk, NY, USA), with the significance level set at *p* ≤ 0.05. The results of the HPLC/MS/MS analysis are presented in Table 1, while the results of ICP/MS are shown in Supplementary Materials Table S1.

Statistical multivariate analysis for untargeted metabolomics was applied to the data matrix combining NMR and HPLC-MS/MS metabolites. Unsupervised PCA and supervised PLS-DA were applied to the entire log-transformed dataset after centering and auto-scaling. The multivariate analysis was carried out using Unscrambler 10.5 software (CAMO, Oslo, Norway). In order to identify a statistically significant variation of the single variables between the two years considered, a Shapiro–Wilk test was applied to define the normality of distribution and then a paired Student’s *t*-test was applied, using Sigmaplot 12.0 software (Systat Software, Inc., San Jose, CA, USA). A *p*-value of 0.05 was considered a threshold for statistical significance.

## 3. Results and Discussions

In Italy, a lockdown due to the COVID-19 pandemic began on 9 March 2020. During this period, the schools were closed and working from home was promoted where possible; travel for unnecessary reasons, sports activities, demonstrations and events was prohibited; non-essential retail commercial activities, catering services, religious celebrations and gatherings of people in public places were suspended.

In addition to the various social, economic and political consequences, the lockdown effects were also evident at the environmental level, especially in terms of a drastic decrease in vehicular traffic, visible above all in large urban centers, such as Rome.

**Figure 1:**
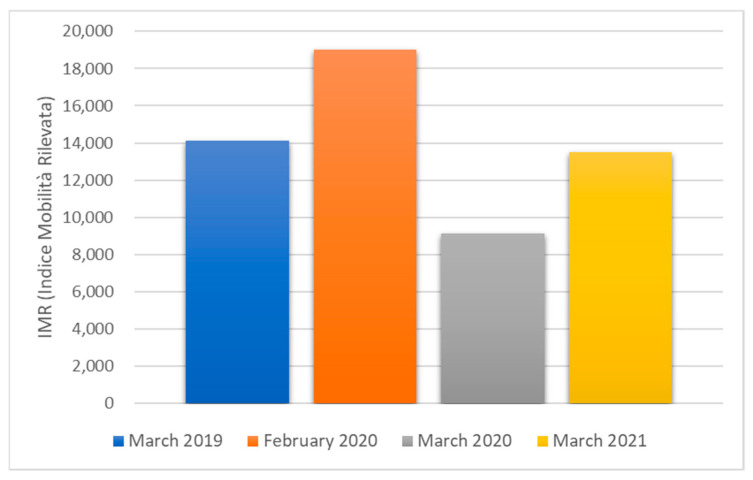
Average monthly traffic data in Italy in March 2019, February and March 2020, and March 2021. IMR (Indice Mobilità Rilevata) is a mobility index expressed as the mean number of vehicles/day, calculated by ANAS S.p.A.

Figure 1 shows the IMR (detected mobility index) recorded by ANAS S.p.A. (the national road agency), which compares the average monthly traffic data in Italy in March 2019, February and March 2020, and March 2021 [65,66].

The restrictive lockdown measures reduced the IMR in the Lazio region (where Rome is located) from February to March 2020 of 36%. The data concerning the month of March 2021 show how the restoration of normal activities brought the mobility index back to a value higher of 48% compared to March 2020.

In order to evaluate the decrease in the airborne concentrations of benzene and PM2.5, the data from the air quality monitoring network of ARPA (the regional agency for environmental protection) of Lazio were used, and in particular those provided in the online weekly bulletins [67,68,69].

**Figure 2:**
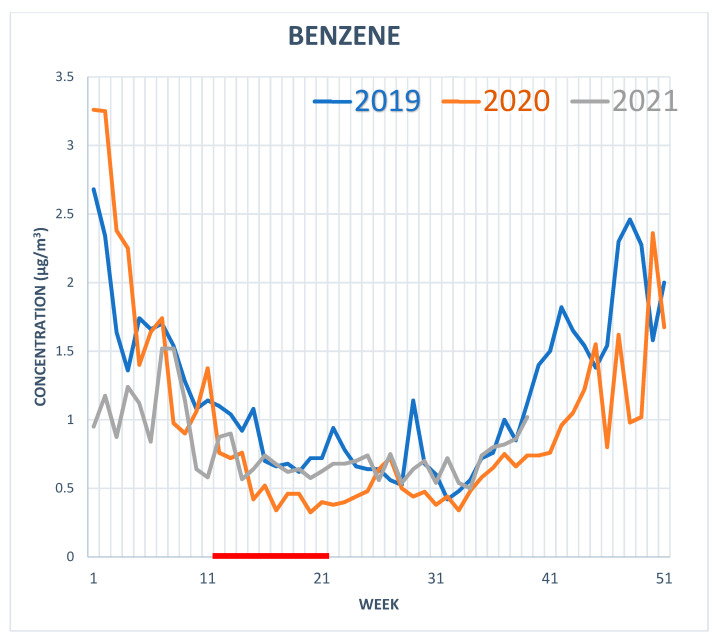
Weekly trend of the concentration of airborne benzene in the years 2019, 2020 and 2021. The weeks 11–21, marked in red, are the lockdown weeks in year 2020.

The weekly values of benzene and PM2.5 airborne concentrations measured by monitoring devices located in five different areas of Rome were averaged. Figure 2 shows the weekly trend of the concentration of airborne benzene in the years 2019, 2020 and 2021. A seasonal decreasing trend can be observed for all the three years, from week 10 to week 30, but the values recorded for the weeks of the lockdown (week 11 to 21, marked in red in the figure) show a reduction in benzene emissions of about 25% compared to the previous year (2019) and of 40% compared to the next (2021), while for PM2.5, there were no significant changes (data not shown).

It can be observed that, while the reduction in benzene concentration is lower but consistent with the decrease in road traffic, for PM2.5 there was not a similar reduction, as other important emission sources have to also be considered, such as civil heating. No decrease in PM2.5 could also result from enhanced atmospheric oxidation during the COVID-19 lockdown [7,10].

The results of the targeted metabolomics, obtained using HPLC-MS/MS, are reported in Table 1 both for the years 2020 and 2021. The values are expressed as a ratio including the urinary creatinine concentrations of the same samples.

### 3.1. Targeted Metabolomics: Determination of Urinary Biomarkers

The urinary concentration values found for the volunteers for each metabolite in 2020 and 2021 were log-transformed and compared using a *t*-test. The statistically significant differences were indicated with an asterisk (*p* < 0.01).

The analysis of the concentrations of the metabolites SPMA and 6-OHNPy shows that their average value in 2020 is about 80% of that found in 2021, while the metabolite of pyrene, 1-OHPy, is about 70% in 2020 compared to 2021, all with a statistically significant difference.

The reduction in the exposure biomarkers in 2020 compared to 2021 reflects, albeit to a lesser extent, both that measured by ARPA Lazio for benzene (−40%) and by ANAS in relation to road traffic in Lazio (−36%). In this regard, it is important to remember that both benzene and PAHs, being produced by the combustion of organic products, can also be generated from indoor sources, such as the use of incense, cooking food and active and passive cigarette smoke, contributing to the production of the studied metabolites.

Examining the effect biomarkers, a highly significant difference was found for all the three measured biomarkers, with a reduction of about 40% for 8-oxoGuo and 8-oxodGuo in 2020 compared to 2021. As regards 8-oxoGua, this biomarker seems to be more sensitive to the exposure variations than the other two, with a reduction of around 60% in 2020 compared to 2021. However, all the biomarkers of oxidative stress were affected by the lockdown more strongly than the dose biomarkers of the considered pollutants.

### 3.2. Determination of Urinary Elements

Supplementary Materials Table S1 shows summary statistics for the creatinine-adjusted urinary metal concentrations at the two monitoring periods. Be, Bi and Mn levels were found to be lower than those of LOD in most or all cases (50% for Bi and 100% of Be and Mn). These elements were excluded from the statistical elaboration. The highest concentrations (>80 mg/g creatinine) were observed for essential elements, such as Ca, K, Mg and Na, while among the toxic or potentially toxic elements, the highest concentrations were obtained for As (mean = 122 or 54 μg/g creatinine in 2020 or 2021, respectively), Cu (mean  =  30 or 40 μg/g creatinine in 2020 or 2021, respectively) and Li (mean  =  27 or 21 μg/g creatinine in 2020 or 2021, respectively). Comparing the cohort at the two time measurements, the analysis showed a generally significant reduction during lockdown year for B, Co, Cu and Sb; Tl and Zn also decreased, but not significantly. We found that B was positively associated with Ni (rho  =  0.713, *p*  <  0.01) and Rb (rho  =  0.706, *p*  <  0.01). Cobalt does not show high correlations with any of the analyzed elements. Cu, Ni and Sb were highly correlated to each other (Spearman correlation ranging from 0.712 to 0.797, *p* <  0.01). Sb was also positively associated with Cr (rho  =  0.723, *p*  <  0.01) and Fe (rho  =  0.704, *p*  <  0.01), indicating a possible common source of exposure. Metals of concern for brake lining emissions include Cd, Cr, Cu, Ni, Pb, Sb and Zn [70]. Previous studies have shown significant contributions of road traffic to the Sb and Cu content of PM due to metal emissions from brake linings [34]. Brake lining wear has been considered to be responsible for 99% and 90% of airborne Sb and Cu, respectively [71]. Additionally, the link between physical activity and increased metal excretion was studied [72,73]. Although sweat is the most important way of the excretion of trace metals during physical exercise [74], it is possible to assume that more intense breathing during physical activity may lead to a greater inhalation of elements present in the air and, consequently, to an increase in their urinary levels. A sedentary lifestyle and reduced vehicular traffic may have caused lower urinary Cu and Sb levels during the 2020 lockdown. However, the difference in B and Co levels between the periods during and after the lockdown could be linked to different eating habits. Fruit-based beverages and products, tubers, legumes, and water could contribute a major portion of the dietary B vitamin [75]. Immune function and the metabolism of vitamin D, Ca, Cu, Mg and estrogen have been proposed as being related to the functioning of B vitamin in humans [75]. Cobalt is an essential trace element that is mainly known as a component of vitamin B12, which serves as a cofactor in the synthesis of methionine and the metabolism of folates and purines [75,76,77]. Good food sources of Co include fish, green leafy vegetables and cereals [78,79].

### 3.3. Untargeted Metabolomics and Multivariate Analysis

A representative urinary ^1^H NMR spectrum is shown in Supplementary Materials Figures S1–S3. Thirty-five metabolites, including creatinine (used as a normalizing factor), were identified and quantified from the ^1^H NMR urine spectra of a subgroup of 34 matched subjects in 2020 and 2021. The list of the identified metabolites with the relative chemical shifts of the resonances is reported in Supplementary Materials Table S2.

The data matrix included, as variables (in columns), 35 urinary NMR metabolites, 6 urinary effect biomarkers (8-oxoGua, 8-oxoGuo, 8-oxodGuo, 3-NO_2_Tyr, 5-MeCyt and Cotinine), 6 urinary dose biomarkers (namely SPMA, 1-OHPy, 6-OHNPy, 3-OHBaP, 1-OHNAP and 2-OHNAP) and 28 urinary elements. All the concentrations were log-transformed before applying the multivariate and univariate analysis performed on 29 pairs of matched samples (mean age of 48.76 ± 16.16 years), among which 14 were females and 15 were males.

**Figure 3:**
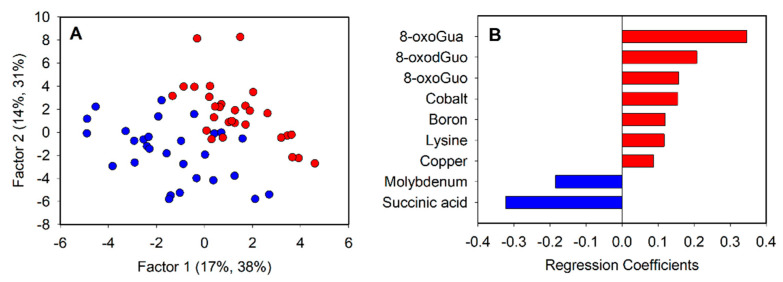
PLS-DA score plot (**A**) and significant regression coefficients (**B**). Blue represents 2020 and red 2021.

Firstly, we performed an explorative PCA for all the subjects (data not shown) to appreciate the possible spontaneous separation between urinary metabolic profiles in 2020 and 2021. Since no spontaneous separation was observed, we decided to analyze the data matrix by PLS-DA in order to identify the effects of the lockdown on the urinary metabolic profiles, as shown in Figure 3.

The PLS-DA (Figure 3A) showed a good discrimination model: the validation results indicate a R^2^ = 0.87 and Q^2^ = 0.49. Furthermore, eight metabolites were identified as significantly relevant for the discrimination based on the regression coefficient values (Figure 3B). In particular, the Lys levels of 8-oxoGuo, 8-oxoGua, 8-oxodGuo, B, Co and Cu were higher in 2021 (red color), while succinic acid and Mo were higher in 2020 (blue color).

**Figure 4:**
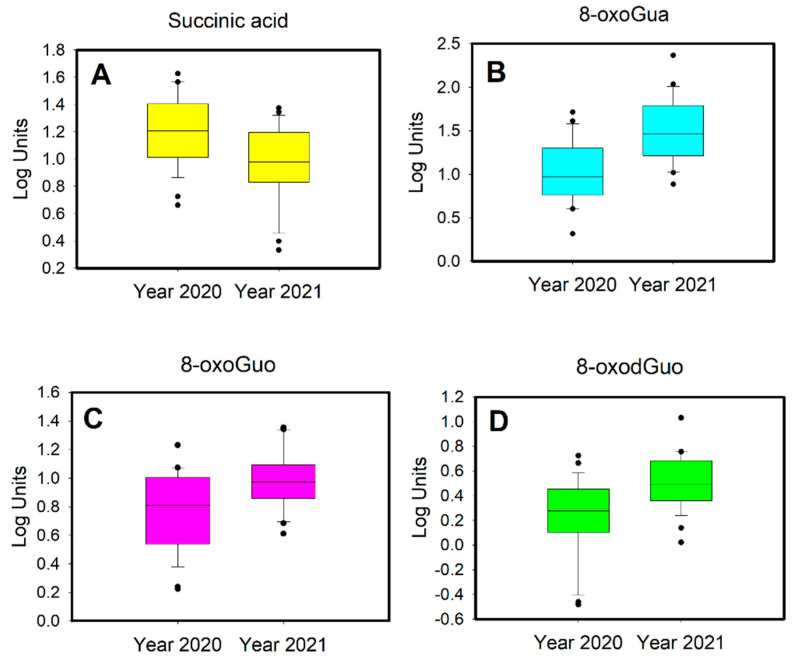
Boxplots of (**A**) succinic acid, (**B**) 8-oxoGua, (**C**) 8-oxoGuo and (**D**) 8-oxodGuo.

**Figure 5:**
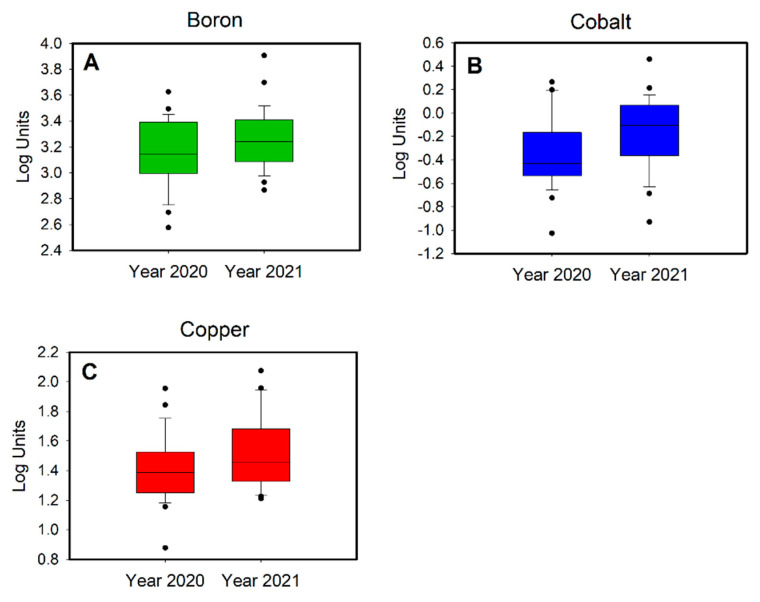
Boxplots of (**A**) boron, (**B**) cobalt and (**C**) copper.

We performed a paired Student’s *t*-test on the metabolites that were significant for the PLS-DA model. We report only the metabolites that have statistically significant differences between 2020 and 2021, as indicated in the boxplots in Figure 4 and Figure 5.

The untargeted metabolomic analysis confirms the results of the targeted HPLC-MS tandem analysis regarding oxidative stress biomarkers, which were higher in 2021 than in 2020. In addition, the increase in succinic acid excretion in 2020 compared to 2021 is highlighted. Succinic acid is an intermediate product of the Krebs cycle. It has recently been observed that succinic acid is able to fuel inflammation and modify cell metabolism [80]. This acid is excreted by macrophages in the extracellular fluids of inflamed tissues; inflammation is favored by a diet rich in sugars, salt and foods containing succinic acid. Furthermore, according to a further study, an increase in urinary levels of succinic acid is due not only to diet but also to a sedentary lifestyle [81].

The increase in the urinary concentration of succinic acid in 2020 is probably attributable to a different diet and a more sedentary lifestyle during the lockdown period.

This study has some limitations. Firstly, the low number of subjects based on the voluntary participation due to the lockdown situation. Secondly, the same subjects had to be contacted one year later for the second sample, causing some dropouts (n = 9). Another limitation is the fact that it was not possible to perform the indoor air monitoring in the houses of the volunteers, due to the reduced mobility of all the Rome residents. In addition, more metabolomic studies with larger sample sizes are needed to identify air pollution components linked to adverse health effects.

## 4. Conclusions

The lockdown scenario was a unique condition, which made possible to directly measure the effects of a reduced exposure to traffic pollution for a long period, on the same group of subjects.

The reduction in the dose exposure biomarkers of benzene and PAHs in 2020 compared to 2021 reflects the trend registered both by ARPA Lazio for airborne benzene concentrations and by ANAS in relation to road traffic in Lazio, Italy.

Lower levels of the urinary oxidative stress biomarkers 8-oxoGuo, 8-oxoGua and 8-oxodGuo were also observed in 2020 with respect to 2021. These three biomarkers were more strongly affected by the lockdown than the benzene and PAH biomarkers: in fact, these pollutants are also produced by the combustion of organic products and, therefore, can be generated from indoor sources that were not influenced by the lockdown, such as wood heating, use of incense, food cooking and active and passive cigarette smoke, contributing to their release in the atmosphere.

Elementary analysis showed a generally significant reduction in B, Co, Cu and Sb during the lockdown year. Indeed, the higher airborne levels of Sb and Cu during 2021 could be associated with a greater vehicular traffic, particularly brake lining wear. A sedentary lifestyle and less vehicular traffic may have also caused the lower urinary Cu and Sb levels during the 2020 lockdown.

The untargeted metabolomic analysis identified succinate, an intermediate of the Krebs cycle, as being lower in 2021, possibly due to a different diet and a more sedentary lifestyle during the 2020 lockdown period.

The results show a significant reduction in the traffic pollution biomarkers in Rome citizens during the lockdown period, and an even stronger reduction in oxidative stress, linked not only to the reduced traffic exposure, but also to a different lifestyle.

This study demonstrates that urban pollution due to traffic is only partly responsible for oxidative stress in the citizens, and that other factors also have a role. We think that it would be important to include the determination of effect biomarkers in the chemical exposure studies. In addition, our results enhance the importance of information and communication about a healthy lifestyle for the prevention of cancer diseases.

## Figures and Tables

**Table 1:** Results of targeted metabolomics expressed in µg/g of creatinine on 47 pairs of matched samples. Asterisks indicate statistical significance (*p* < 0.01).

Analyte	8-oxoGua	8-oxoGuo	8-oxodGuo	3-NO_2_Tyr	5-MeCyt	SPMA	1-OHPy	6-OHNPy	3-OHBaPy	1-OHNAP	2-OHNAP
	YEAR 2020										
mean	15.99 *	6.73 *	2.26 *	33.91	11.33	0.31 *	0.05 *	>LOD	0.04	0.43	4.65
SD	14.54	4.31	1.25	27.60	6.99	0.20	0.04	>LOD	0.04	0.76	6.12
median	9.72	5.36	2.21	21.74	9.89	0.23	0.04	>LOD	0.02	0.27	2.89
5th perc	4.02	2.14	0.36	12.04	5.03	0.13	0.02	>LOD	0.01	0.01	1.03
95th perc	47.85	15.46	4.38	103.52	21.85	0.73	0.13	0.01	0.14	1.63	12.85
min	2.07	1.67	0.09	7.96	4.33	0.09	0.01	>LOD	0.01	0.01	0.52
max	64.46	19.54	5.31	126.20	43.13	0.94	0.18	>LOD	0.17	4.62	39.22
	YEAR 2021										
mean	46.50	10.60	3.64	32.95	12.72	0.39	0.07	>LOD	0.03	0.46	6.08
SD	43.98	5.61	1.84	24.81	8.05	0.23	0.03	>LOD	0.03	1.15	6.80
median	28.80	8.66	3.14	23.11	9.89	0.31	0.06	>LOD	0.02	0.12	3.66
5th perc	6.89	4.52	1.42	14.08	5.12	0.14	0.03	>LOD	0.01	0.01	0.76
95th perc	118.19	22.39	5.98	69.41	30.86	0.83	0.13	0.01	0.07	1.82	19.48
min	4.43	3.89	1.05	3.17	3.68	0.14	0.02	>LOD	0.01	0.01	0.01
max	232.30	24.50	10.74	142.61	38.48	1.07	0.16	0.02	0.18	7.43	37.32

## Data Availability

The data presented in this study are available within this article. Further inquiries may be directed to the authors.

## Supplementary Materials

The following supporting information can be downloaded at: https://www.mdpi.com/article/10.3390/toxics10050267/s1, Table S1: Descriptive statistic of 28 urinary elements (µg/g creatinine) on 29 pairs of matched samples; Figure S1: ^1^H NMR spectrum of urine t; Figure S2: ^1^H NMR spectrum of urine, spectral region of 1–4 ppm; Figure S3: ^1^H NMR spectrum of urine, spectral region of 6.5–9.5 ppm; Table S2: ^1^H NMR assignment of urinary metabolites.
