# Supplementary material for: Biomonitoring of Exposure to Urban Pollutants and Oxidative Stress during the COVID-19 Lockdown in Rome Residents
Source: Toxics. 2022 May 21;10(5):267. doi: 10.3390/toxics10050267 (PMC9143243; doi:10.3390/toxics10050267)
Supplement: Supplementary file 1 [file toxics-10-00267-s001.zip › toxics-1722362-supplementary.pdf]

Effect of the 2020 lockdown during Covid-19 pandemic on the biomarkers of exposure to urban pollutants and oxidative stress in Rome residents

Flavia Buonaurio[1], Francesca Borra[1], Daniela Pigini[2], Enrico Paci[2], Mariangela Spagnoli[2], Maria Luisa Astolfi[1], Ottavia Giampaoli[3,4], Fabio Sciubba[3,4], Alfredo Miccheli[3,4], Silvia Canepari[3], Carla Ancona[5] and Giovanna Tranfo[2, *]

[1] *Department of Chemistry, Sapienza University of Rome, Italy*
[2] *Department of Occupational and Environmental Medicine, Epidemiology and Hygiene, INAIL, Monte Porzio Catone, Rome, Italy*
[3] *Department of Environmental Biology, Sapienza University of Rome, Italy*
[4] *NMR-based Metabolomics Laboratory (NMLab), Sapienza University of Rome, Italy*
[5] *Department of Epidemiology, Lazio Regional Health Service, Rome, Italy*

* Correspondence: g.tranfo@inail.it; Tel: +39-694181436

Supplementary Table S1. Descriptive statistic of 28 urinary elements (μg/g creatinine) on 29 pairs of matched samples

Elements			year 2020					year 2021					
	LOD[a]	LOQ[b]	mean	SD[c]	median	min	max	mean	SD[c]	median	min	max	p-value[d]
**As**	2	8	122	289	36	4	1524	54	73	23	2	311	ns
**B**	30	120	**1650**	**910**	1390	377	4220	**2150**	**1500**	1740	736	8060	*
**Ba**	3	9	4.9	3.3	3.7	0.6	13.2	4.9	4.7	3.7	0.5	25.1	ns
**Be**	0.02	0.08	<0.02	-	<0.02	<0.02	<0.02	<0.02	-	<0.02	<0.02	<0.02	nd
**Bi**	0.01	0.03	0.02	0.05	0.01	<0.01	0.25	<0.01	-	<0.01	<0.01	0.03	ns
**Ca**	500	2000	198000	120000	172000	36086	504000	175000	120000	147000	29300	582000	ns
**Cd**	0.04	0.1	0.22	0.16	0.20	<0.04	0.69	0.24	0.16	0.20	<0.04	0.70	ns
**Co**	0.05	0.2	**0.56**	**0.45**	0.37	0.09	1.85	**0.87**	**0.57**	0.79	0.12	2.90	*
**Cr**	0.2	0.6	5.2	3.5	4.1	1.0	15.6	5.7	4.6	4.2	1.2	24.5	ns
**Cs**	0.01	0.03	19	21	13	3	94	25	29	14	4	105	ns
**Cu**	0.4	1	**30**	**18**	24	8	90	**40**	**27**	29	16	119	*
**Fe**	1	4	18	12	13	3	48	18.6	8.5	16	7	38	ns
**Hg**	0.03	0.09	0.62	0.41	0.49	0.13	1.71	0.69	0.48	0.51	0.14	2.14	ns
**K**	700	2500	1320000	830000	1010000	158000	3780000	1580000	900000	1310000	348000	4400000	ns
**Li**	0.04	0.1	27	37	13	3	182	22	19	14	3	71	ns
**Mg**	5	20	92820	43900	81100	37900	225000	90600	45200	83200	35300	241000	ns
**Mn**	5	20	<5	-	<5	<5	<5	<5	-	<5	<5	<5	nd
**Mo**	0.6	2	53	44	39	11	178	44	34	38	7	148	ns
**Na**	30	90	2550000	1900000	1860000	547000	950000	2460000	1700000	2080000	51700	8760000	ns
**Ni**	0.6	2	6.2	3.4	5.5	1.7	17.8	6.5	3.1	5.7	2.7	15.5	ns
**Pb**	0.04	0.1	1.2	1.1	0.8	0.2	4.4	1.0	0.5	1.0	0.3	2.3	ns
**Rb**	0.07	0.2	967	520	857	238	2050	1160	680	995	256	2740	ns
**Sb**	0.01	0.04	**6.9**	**5.1**	4.7	0.8	21.7	**8.9**	**5.7**	7.6	1.2	25.6	*

**Se**	1	3	34	16	33	15	79	33	16	29	16	76	ns
**Sn**	0.04	0.1	0.8	2.0	0.3	0.1	10.4	0.4	0.2	0.4	0.1	1.0	ns
**Sr**	0.5	2	199	100	168	59	538	195	120	184	40	619	ns
**Te**	0.2	0.5	15	33	5	0.4	179	13	16	7	2	76	ns
**Tl**	0.001	0.005	6.5	7.5	4.2	0.2	26.6	9.9	9.7	6.4	0.6	39.5	ns
**V**	0.3	1	1.6	2.4	0.3	0.3	9.0	0.9	1.5	0.3	0.3	6.5	ns
**Zn**	3	9	345	200	315	88	1160	394	200	323	91	791	ns

[a] LOD, limit of determination

[b] LOQ, limit of quantification

[c] SD, standard deviation

[d] Paired Student's t test was applied: "nd" = not determined; "ns" = not significant at $p > 0.05$; "*" = significant at $p < 0.05$. Numbers in bold in the same row indicate significant differences ($p < 0.05$).

Supplementary Figure S1. ^{1}H NMR spectrum of urine

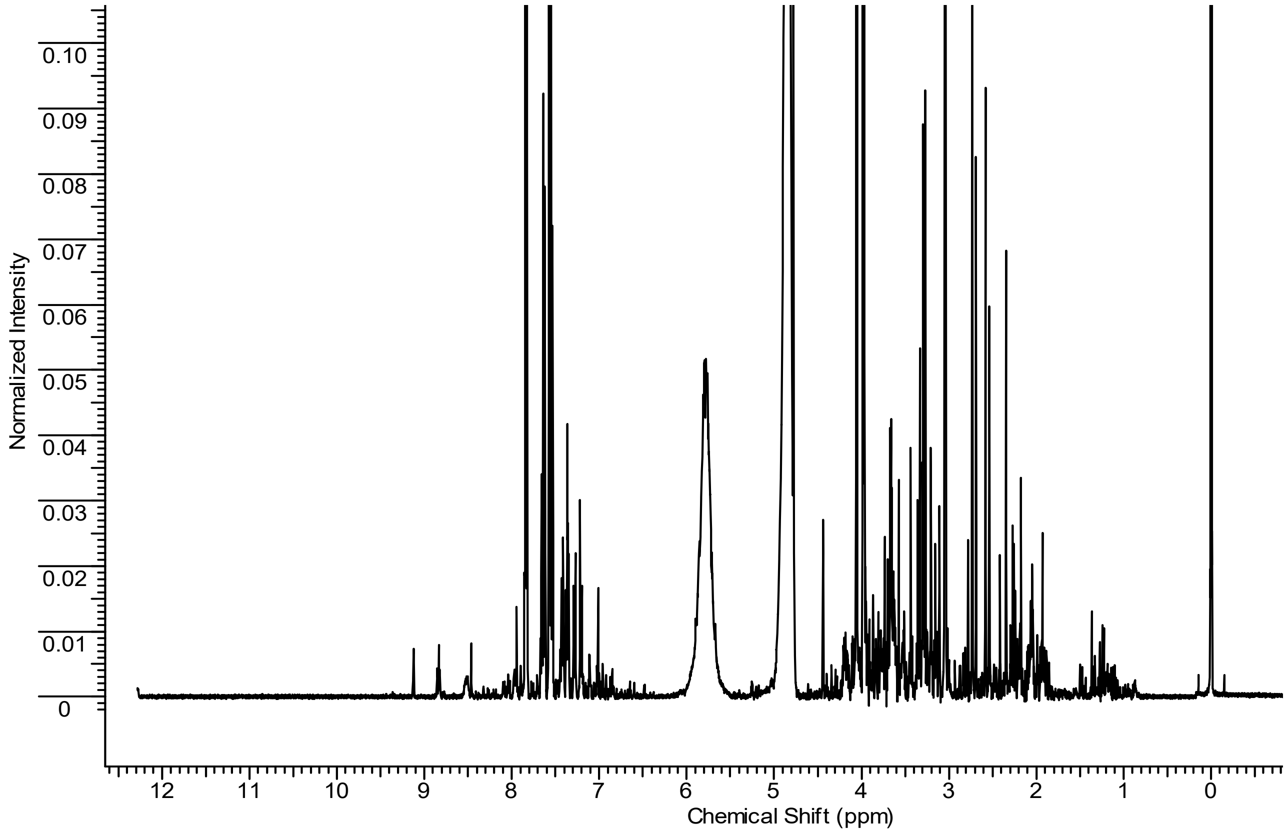

Supplementary Figure S2. ^{1}H NMR spectrum of urine, spectral region 1 – 4 ppm.

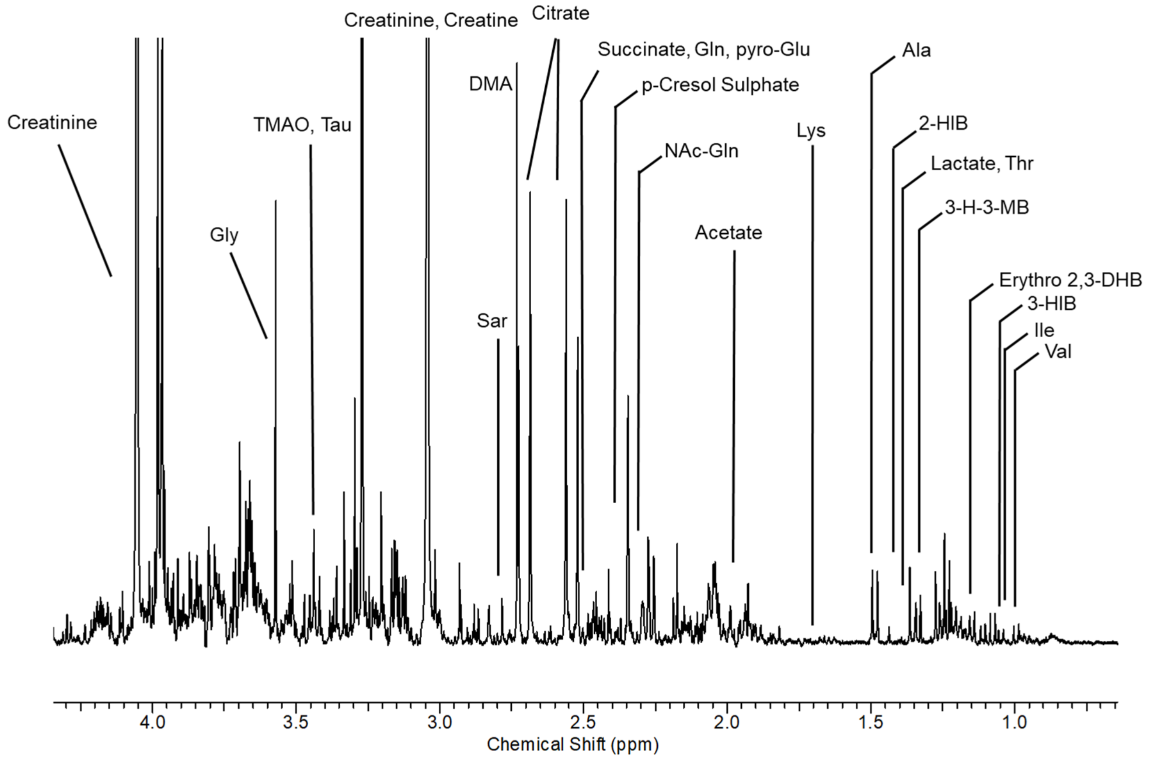

Supplementary Figure S3. ^{1}H NMR spectrum of urine, spectral region 6.5 – 9.5 ppm.

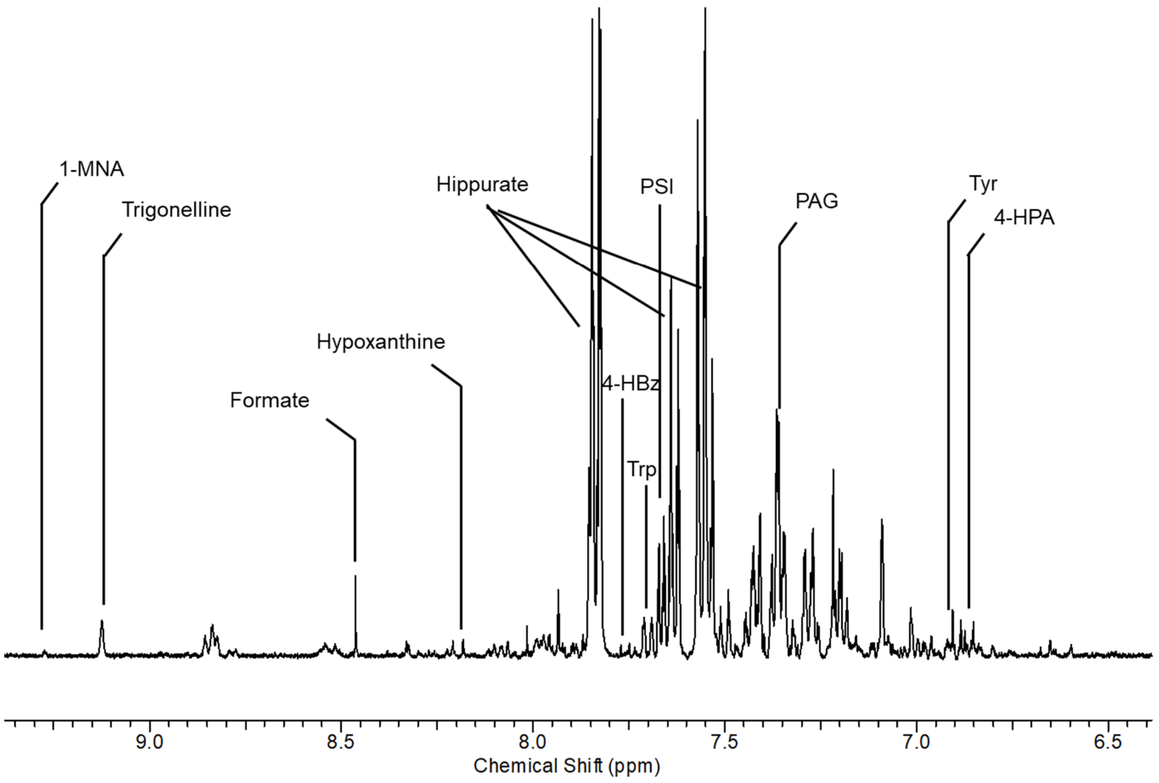

Supplementary Table S2. ^{1}H NMR assignment of urinary metabolites.

bs: broad singlet; d: doublet; dd: double doublet; m: multiplet; pd: pseudo doublet; q: quartet; s: singlet, t: triplet.

Metabolite	Structure	^{1}H δ ppm	Multiplicity	Assignment
1. Valine (Val)	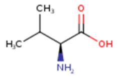	0.99<br>**1.05**	d<br>**d**	CH_3<br>**CH_3'**
2. Isoleucine (Ile)	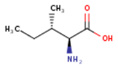	0.92<br>**1.01**<br>1.99	t<br>**d**	CH_3<br>**CH_3**<br>CH
3. 3-Hydroxyisobutyrate (3-HIB)	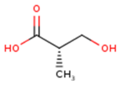	**1.07**<br>2.49<br>3.54<br>3.71	**d**	**CH_3**
4. Erythro-2,3-dihydroxybutyrate (Erythro-2,3-DHB)	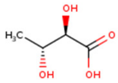	**1.11**<br>4.19	**d**<br>m	**CH_3**<br>CH
5. 3-Hydroxy-3-methylbutyrate (3-H-3-MB)	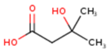	**1.27**	**s**	**CH_3, CH_3'**
6. Lysine (Lys)	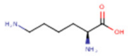	1.48<br>**1.71**<br>1.89<br>3.02<br>3.74	m<br>**m**<br>m<br>m<br>m	CH_2<br>**CH_2**<br>CH_2<br>CH_2<br>CH
7. Lactate	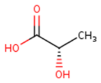	**1.33**<br>4.11	**d**<br>q	**CH_3**<br>CH
8. Threonine (Thr)	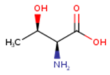	**1.33**<br>3.59<br>4.26	**d**<br>d<br>m	**CH_3**<br>α-CH<br>β-CH
9. 2-Hydroxyisobutyrate (2-HIB)	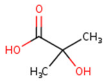	**1.36**	**s**	**CH_3, CH_3'**
10. Alanine (Ala)	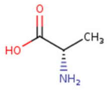	**1.49**<br>3.78	**d**<br>q	**CH_3**<br>α-CH
11. Acetate	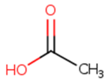	**1.93**	**s**	**CH_3**

12. N-acetylglutamine (NAcGln)	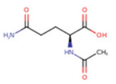	1.95<br>2.12<br>**2.27**<br>4.18<br>7.97	 <br>**m**<br><br>bs	 <br>**CH_2**
13. Pyroglutamate (pyro-Glu)	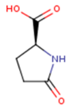	2.03<br>**2.40**<br>2.50<br>4.19	m<br>**m**<br>m<br>dd	CH_2<br>**CH**<br>CH<br>CH
14. Glutamine (Gln)	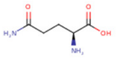	2.13<br>**2.46**<br>3.78	m<br>**m**<br>t	CH_2<br>**CH_2**<br>CH
15. p-Cresol sulphate	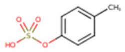	**2.35**<br>7.21<br>7.28	**bs**<br>dd<br>dd	**CH_3**<br>CH, CH'<br>CH, CH'
16. Succinate	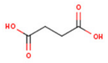	**2.41**	**s**	**CH_2, CH_2'**
17. Citrate	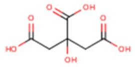	**2.54**<br>2.69	**d**<br>d	**CH, CH'**<br>CH, CH'
18. Dimethylamine (DMA)	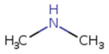	**2.73**	**s**	**CH_3, CH_3'**
19. Sarcosine (Sar)	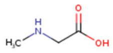	**2.78**	**s**	**CH_3**
20. Creatine	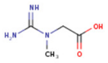	**3.05**<br>3.95	**s**<br>s	**CH_3**<br>CH2
21. Creatinine	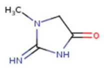	3.03<br>**4.05**	s<br>**s**	CH_3<br>**CH_2**
22. Trimethylamine N-Oxide (TMAO)	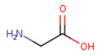	**3.27**	**s**	**CH_3**<br>CH_2
23. Taurine (Tau)	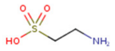	3.27<br>**3.43**	t<br>**t**	CH_2<br>**CH_2**
24. Glycine (Gly)	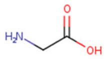	**3.57**	**s**	**CH_2**
25. 4-Hydroxyphenylacetate (4-HPA)	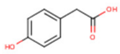	**6.87**<br>7.17	**dd**<br>dd	**CH,CH**<br>CH,CH

26. Tyrosine (Tyr)	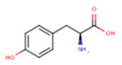	**6.90**<br>7.18	**dd**<br>dd	**2-CH,6-CH**<br>3-CH,5-CH
27. 4-Hydroxybenzoate (4-HBz)	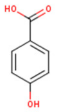	6.97<br>**7.76**	dd<br>**dd**	2-CH,6-CH<br>**3-CH,5-CH**
28. Tryptophan (Trp)	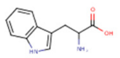	7.20<br>7.27-<br>7.29-<br>**7.50**<br>7.70	-<br>-<br>-<br>**pd**<br>pd	3-CH<br>2-CH<br><br>**5-CH**<br>4-CH
29. Phenylacetylglycine (PAG)	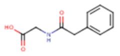	**7.36**<br>7.39<br>7.42	**m**<br>m<br>m	3,5-CH<br>4-CH<br>2,6-CH
30. Hippurate (Hipp)	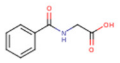	3.97<br>**7.55**<br>7.64<br>7.83	d<br>**m**<br>m<br>m	CH2<br>**3,5-CH**<br>4-CH<br>2,6-CH
31. Pseudouridine (PSI)	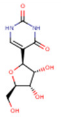	**7.67**	**bs**	**CH**
32. Hypoxanthine (HX)	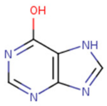	**8.19**<br>8.21	s<br>s	2-CH<br>7-CH
33. N1-Methyl-2-pyridone-5-carboxamide (2PY)	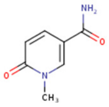	3.64<br>6.67<br>7.98<br>**8.33**	s<br>d<br>d<br>**dd**	N-CH_3<br>3-CH<br>4-CH<br>**6-CH**
34. Formate	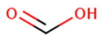	**8.46**	**s**	**CH**
35. U01		8.06<br>8.54<br>**8.78**	 <br>**d**	 <br>**6-CH**
36. Trigonelline (Trig)	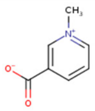	4.44<br>8.08<br>8.84<br>**9.12**	s<br>m<br>m<br>**s**	N-CH_3<br>5-CH<br>4,6-CH<br>**2-CH**
37. 1-MNA	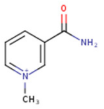	8.17<br>8.89<br>8.96<br>**9.28**	t<br>d<br>d<br>**s**	5-CH<br>4-CH<br>6-CH<br>**2-CH**
